# Foreign Substances in the Body

**Published:** 1882-07

**Authors:** T. P. McElreath

**Affiliations:** Wo dbury, Ga.


### FOREIGN SUBSTANCES IN THE BODY—REPORT OF TWO CASES.

By T. P. McELREATH, M.D., Wo dbury, Ga.

*Case I.*—During the month of August, 1881, Dr. D——, of this place, consulted me concerning his little boy, 7 years of age, stating that he had a mucous discharge, sometimes mixed with blood, from his left nostril, and that it had continued about ten months. He could assign no cause for the trouble unless it was the result of ulceration or polypi. After hearing his statement I agreed that this was very likely the cause, and made no examination myself, but suggested that he carry the little boy to a surgeon who was better prepared than I to make an examination of the nasal cavity. He adopted this advice, and the surgeon made a searching examination and diagnosed "ulceration of the posterior nares." He put him upon a routine of washing and drugging suitable for the supposed malady, but without improvement. The mucosanguinous discharge continued, and pain about the nose was sometimes complained of. The little fellow made a peculiar whistling noise as he breathed when asleep, and he was observed to sleep with his mouth open. But he attended school, ran about and played as other children, and seemed to suffer very little inconvenience. This state of things continued until a few days ago, when, by the aid of a bright sun light, his father saw something, he knew not what, high up in the nasal cavity, and seizing it with a small forceps, he with some difficulty extracted a piece of hard, almost dry weed, a little over two inches in length and one eighth of an inch in diameter. Now, ten days after the extraction of this foreign body, the little boy is well. He has carried this "thorn in the flesh" for eighteen months.

*Case II.*—Last November Mr. G. presented his child, about six months of age, to have a needle removed, which had been driven into the arm by a fall upon it. Dr. J. T. Dixon and myself examined the case.

We found the needle had entered the forearm on the outer side of the ulna about one and a half inches from the wrist, and ranged from below upwards by the side of the radius. As the child seemed to suffer no inconvenience we declined making an exploratory incision (the needle having entirely disappeared), but advised the father to wait until the needle made its appearance under the skin at some point, or should cause some trouble to the child—then to bring it back. Accordingly, a few days since Mr. G. brought his child again, stating that he could "see" the needle near the elbow.

We now detected the needle lying under the skin just over the tuberosity of the radius—a little below the elbow joint and just on the opposite side of the arm where it entered, and three or more inches higher up the arm.

After making a slight incision in the skin it required a good deal of patient manipulation to cause the needle to approach the opening. Occasionally it would disappear among the muscles and tendons and seem to be quite gone, but after a time a common sewing needle—No. 6—very rusty, with the point broken off, but yet measuring a little over one inch in length, was extracted from amongst the muscles where it had been burrowing for the last seven months. The child did not suffer at all from its presence.

